# Mining Knowledge of Respiratory Rate Quantification and Abnormal Pattern Prediction

**DOI:** 10.1007/s12559-021-09908-8

**Published:** 2021-07-10

**Authors:** Piotr Szczuko, Adam Kurowski, Piotr Odya, Andrzej Czyżewski, Bożena Kostek, Beata Graff, Krzysztof Narkiewicz

**Affiliations:** 1 Multimedia System Department, Faculty of Electronics, Telecommunications and Informatics, Gdańsk University of Technology, 80-233 Gdańsk, Poland; 2 Audio Acoustics Department, Faculty of Electronics, Telecommunications and Informatics, Gdańsk University of Technology, 80-233 Gdańsk, Poland; 3 Department of Hypertension and Diabetology, Medical University of Gdansk, 80-210 Gdańsk, Poland

**Keywords:** Granular analysis, Rough sets, k-NN, UMAP, Respiratory pattern analysis, Wavelet analysis (DWT, discrete wavelet transform)

## Abstract

The described application of granular computing is motivated because cardiovascular disease (CVD) remains a major killer globally. There is increasing evidence that abnormal respiratory patterns might contribute to the development and progression of CVD. Consequently, a method that would support a physician in respiratory pattern evaluation should be developed. Group decision-making, tri-way reasoning, and rough set–based analysis were applied to granular computing. Signal attributes and anthropomorphic parameters were explored to develop prediction models to determine the percentage contribution of periodic-like, intermediate, and normal breathing patterns in the analyzed signals. The proposed methodology was validated employing k-nearest neighbor (k-NN) and UMAP (uniform manifold approximation and projection). The presented approach applied to respiratory pattern evaluation shows that median accuracies in a considerable number of cases exceeded 0.75. Overall, parameters related to signal analysis are indicated as more important than anthropomorphic features. It was also found that obesity characterized by a high WHR (waist-to-hip ratio) and male sex were predisposing factors for the occurrence of periodic-like or intermediate patterns of respiration. It may be among the essential findings derived from this study. Based on classification measures, it may be observed that a physician may use such a methodology as a respiratory pattern evaluation-aided method.

## Introduction

**Fig. 1:**
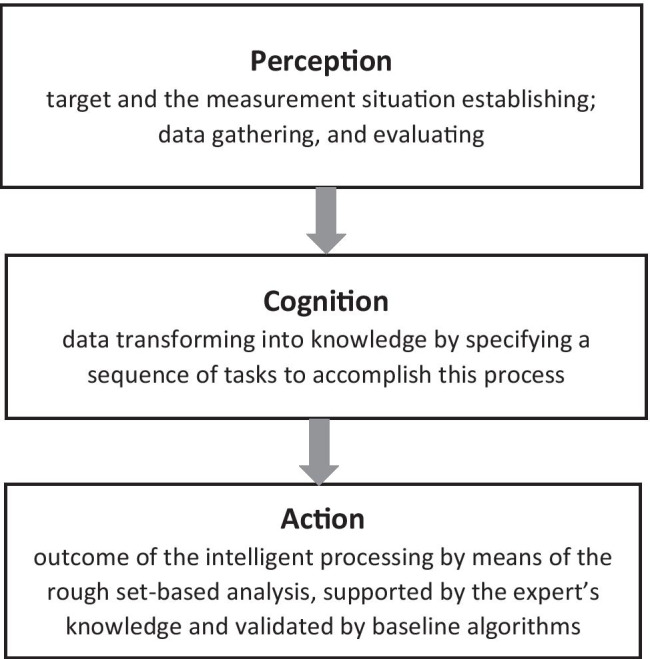
Tri-stage conceptual model applied to respiratory pattern recognition

The aim of this study is threefold. First, it aims to use group reasoning [1–3] to investigate how to handle data comprising health indicators and breathing signal characteristics and the machine learning approach that should be employed. Therefore, in the pre-analytic stage, physicians, diagnosticians, and computer scientists were engaged to discuss several possible ways to manage the collected data. For example, as we deal with real-world data that contain uncertain or incomplete samples, deep learning was dismissed at the early stage of the analysis, as we needed to obtain a better insight into the analysis beyond quantitative assessment. Simultaneously, group reasoning can be treated as a part of the tri-way conceptual reasoning model proposed by Yao [4] and adopted from Nanay [5]. Second, this study aims to follow Yao’s [4] three stages of reasoning: perception, cognition, and action (Fig. 1). Finally, after performing group reasoning, we found that there were many factors collected in diagnostics, which were cost- and time-intensive. Because of COVID-19, determining which should be retained is imperative. Otherwise, data processing may be time-consuming, decisions may be too slow, or the diagnostic pathway may be affected by a specific component contributing to the overall medical context. Consequently, we decided to focus on tri-way reasoning [4], applying granular computing [6–10], and rough sets [6, 11–13] to knowledge mining. Moreover, the approach proposed is validated by a baseline k-nearest neighbor algorithm and UMAP (uniform manifold approximation and projection) visualization.

In this study, we deal with data related to cardiovascular disease (CVD). CVD remains a major killer in the world. Each year CVD causes 3.9 million deaths in Europe, including 1.8 million deaths in the European Union [14]. There is increasing evidence that altered respiration might contribute to the development and progression of CVD. Abnormal respiratory patterns are common in patients with severe conditions, including congestive heart failure (CHF) and obstructive sleep apnea (OSA). The so-called Cheyne–Stokes respiration (CSR) during sleep, presenting as repeating rises and falls in ventilation separated with periods of apnea (cessation of breathing), is a common finding in patients with CHF [15]. In these patients, a similar respiration pattern with apnea (CSR) or without (periodic breathing, PB) was also frequent during the day. Furthermore, it was shown that the cyclical pattern of breathing is a marker of poor outcomes [16]. Diagnosis of OSA is based on the investigation of respiratory patterns during sleep, but it is limited to apnea and hypopnea detection and focuses on the differentiation between obstructive and central apneas [15]. Therefore, the respiratory rate quantification and abnormal pattern prediction deviate from the fine-grained universe in which every bit of information is ordered adequately towards coarse-grained ones as “normal” and “disrupted” respiratory patterns could only roughly be discerned between each other category.

A concept of granularity in medicine has existed for decades. It already appeared among the studies dating back to 1998, when Tange et al. [17] referred to clinical narratives containing free text as “high granularity” segments. Information retrieval from clinical narratives involves several steps: searching for a labeled segment, reading its content, and analyzing it. In the authors’ opinion, physicians can retrieve information better when clinical narratives written in the free text are divided into many small labeled segments, i.e., granules.

Qi et al. defined five types of granules, namely, those induced by objects and attributes, and the ones induced by both objects and attributes simultaneously are seen from different perspectives and levels. Pal discerns three components of granular computing (GrC), i.e., granulation, granules, and computing with granules [6]. In this study, the granularity concept should be understood as a set of objects with descriptions derived from discretization. These granules involve a reduced number of relevant features, resulting in dimensionality reduction. In this sense, clusters or segments formed by granulation are called granules. Therefore, a granule may be defined as a collection of indiscernible entities that are collected according to their similarity, proximity, or functionality regarding given attributes [6, 7, 18].

Biomedical signals are often analyzed with the use of Gabor transform and discrete wavelet transform (DWT) [19−21]. This study aimed to determine the relationship between phase coherence and instantaneous heart rate and respiration. One of the critical points of this study is that PB, in which slow periodic oscillations modulate the regular oscillations corresponding to rhythmic expiration and inspiration, evoked high altitude-induced hypoxia. Also, using signal processing based on wavelet analysis helped to analyze mechanisms underlying respiratory control during hypobaric hypoxia, which is related to genetics and cardiovascular dynamics [22].

Notably, the graphical results of wavelet analyses were treated as clouds and granules when they were first used. However, this concept was not further developed. Also, there were different directions of granularity concepts foreseen and applied to general medicine, medical informatics, cohort selection, risk prediction, and healthcare quality measurement [23, 24]. Notions such as multi-granularity embeddings [23], coarse- or fine-grained objects, were discussed in medical data analysis through granularity principles [24].

Signal analysis employing wavelet analysis was performed “behind-the-scenes” along with physicians’ subjective evaluation, showing another level of granularity according to the adopted Yao’s model [4]. Also, this study gained knowledge from the data collected, processed, and analyzed (ETL, extract–transform–load phase [25]). Roughly, we divided the signal analysis outcome as granules of normal breathing signal patterns, periodic-like signals, pauses, or apneas.

Short-term daytime signal recordings are based on various respiratory belts designed to measure chest diameter changes resulting from breathing. In contrast to the analysis of heart rate and blood pressure variability, respiration pattern assessment is not well developed, and comprehensive methods for full automatic detection of a periodic pattern of breathing are rare. In many previous studies, the main part of respiratory pattern assessment was mostly based on a visual inspection. There are a few examples of using the combination of visual assessment and computerized analysis of the breathing pattern [16, 26, 27]. The outcome of the automatic classification of respiratory signals to detect abnormalities in breathing or breathing cessations is encouraging. Yet, this needs to be further developed.

Thus, there is a clear need to develop novel methods to measure and analyze respiratory variability, especially in healthy individuals and patients with early stages of CVD presenting a spectrum of respiratory pattern alterations, including cyclical behavior, that does not meet the criteria for CSR. These novel methods, especially if combined with parallel assessment of heart rate and blood pressure variability, might provide better insights into cardiorespiratory regulation in health and in disease and induce better prevention and treatment of CVD. Therefore, we propose a new approach to respiratory pattern assessment, namely rough set–based processing of data [11, 12], relying, however, on Yao’s perception–cognition–action tri-level conceptual model [4] as the starting point (Fig. 1). This tri-level conceptual model explains how cognition is needed as an intermediate between perception and action to better apply intelligent data analytics and study human understanding [4]. Moreover, the model basis lies in the data–information–knowledge/wisdom (DIKW) hierarchy, another way of rationalizing in threes [28]. In fact, Yao builds the perception–cognition–action model around machine/system (collection, analysis, and decision), DIKW (data, information, and knowledge/wisdom), and human (perception, cognition, and action) layers.

Our understanding of this model is as follows: the perception layer established the target and the measurement situation — it gathered and evaluated the data. Moreover, at this stage, group reasoning was applied. Cognition is associated with mental processes that involve gaining knowledge and comprehension; that is, showing context and finding answers hidden in large volumes of information transforms data into knowledge by specifying a sequence of tasks to accomplish this process. Finally, action is the outcome of intelligent processing through rough set–based analysis supported by the expert’s knowledge. Accordingly, all steps of the conceptual model are shown in Fig. 1.

**Fig. 2:**
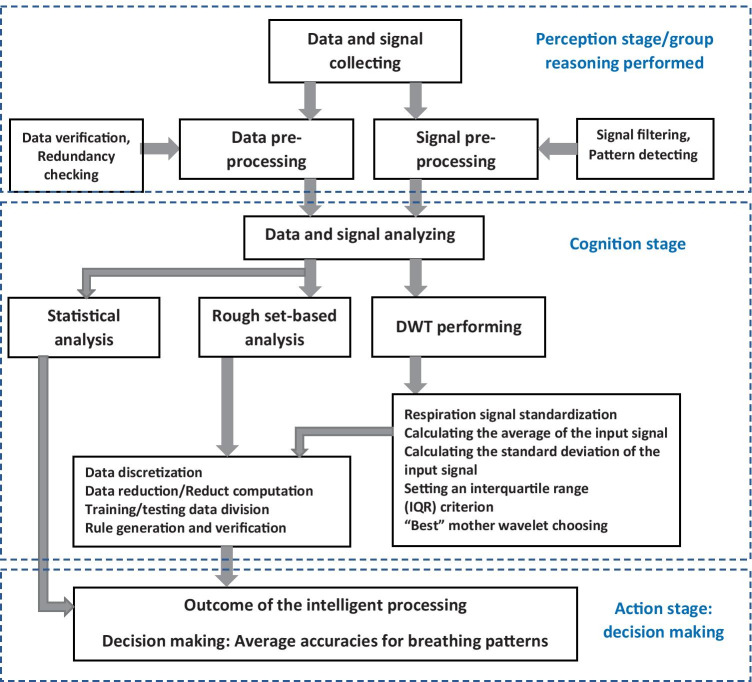
Flowchart of the experiment

Also, we considerably followed the approach proposed by Polkowski and Artiemjew [29], who developed a classifier for coronary heart disease, first using data pre-processing techniques in dealing with the missing values. Second, granular classifier is applied to discover the absence or presence of coronary disease. The flowchart of our performed experiment is presented in Fig. 2.

## Data Analysis

### Study Group

This study complies with the Declaration of Helsinki, and the ethics committee of the Medical University of Gdansk approved its protocol (NKEBN/422/2011). All participants were informed about the merits of the study and signed written consent forms.

The study group comprised 276 subjects (157 men) aged 51.4 ± 11 years. Among them, 151 had a history of hypertension, 28 experienced a transient ischemic attack, 21 were diagnosed with obstructive sleep apnea, and there were 11 diabetic patients. The mean body mass index (BMI) in this group was 28.8 ± 4.9 kg/m^2^, and the waist-to-hip ratio (WHR) was 0.92 ± 0.10.

In each subject, 20-min recordings of respiration were performed in the supine position. All patients were asked to relax, but not to fall asleep. The respiratory belt (Pneumotrace II™), based on a piezoelectric device connected to PowerLab with the LabChart software (ADInstruments, Australia), was used to derive breathing patterns. The sampling rate was 1000 Hz. Using the LabChart software, respiratory tracings were visualized, and breathing patterns were classified by the physician as normal (for a respiratory signal with similar amplitudes), periodic-like (characterized by cyclical behavior of breathing pattern including waxing and waning of amplitude), or intermediate (including various types of the pattern). At this stage, no additional methods for detecting PB (for example, time-varying spectral density analysis) were used.

As in many subjects, breathing patterns changed during recording, and the percentage of a given type of breath in all patients was indicated. The intermediate type of pattern included all cases when the percentage of normal or periodic-like pattern was rated as less than 70%. It should be stressed that all of these classifications were entirely subjective. Among 276 studied subjects, 92 were classified as having a normal breathing pattern, 56 had a periodic-like pattern, and 128 had an intermediate pattern of breathing. Table 1 presents the data collected from the questionnaire forms based on a clinical assessment, which were subsequently completed by respiratory signal analysis and their further evaluation.

**Table 1:** Information about subjects based on clinical assessment

Subject number	Age	Sex	Anthropometric data						Respiratory pattern characteristics						
			Weight [kg]	Height [cm]	Waist [cm]	Hip [cm]	BMI [kg/m^2^]	WHR	Apneas^**^			Percentage contribution of pattern			Pattern’s type by expert^*^
									Number	Number per minute	Mean duration	Periodic-like	Intermediate	Normal	
1	31	M	93.00	182	101	106.0	28.0763	0.9528	0	0	0	30	0	70	2
2	20	F	87.00	164	90	111.0	32.3468	0.8108	0	0	0	0	10	90	1
3	27	M	67.00	174	79	91.0	22.1297	0.8681	0	0	0	0	10	90	1
⋮	⋮	⋮	⋮	⋮	⋮	⋮	⋮	⋮	⋮	⋮	⋮	⋮	⋮	⋮	⋮
273	31	M	127.00	190	116	121.0	35.1801	0.9587	8	0.4002	11.4688	70	30	0	3
274	36	M	105.00	190	104	114.0	29.0859	0.9123	0	0	0	50	40	10	2
275	60	F	115.70	178	118	115.0	36.5169	1.0261	2	0.1	14.695	50	40	10	2
276	45	F	80.00	170	95	100.0	27.6817	0.9500	0	0	0	0	20	80	1

*M* male, *F* female, *BMI* body mass index, *WHR* waist (circumference)-to-hip (circumference) ratio

^*^Pattern type by expert: 1, normal: 2, intermediate: 3, periodic-like

^**^Apneas were defined when there was no respiratory activity lasting longer than three breathing cycles

### Statistical Analysis of Data Acquired

For each parameter (age, weight, height, waist, HIP, BMI, and WHR), significant differences between the obtained results were calculated using the Kruskal–Wallis test. The Kruskal–Wallis test is an extension of the Wilcoxon rank sum test but opposite to it, and it is not limited to two populations [30]. The null hypothesis in this test is the assumption that medium ranks or the medians of the data series are the same. The statistical calculations were performed using MATLAB. The obtained results are presented in Table 2.

**Table 2:** *p*-values from the Kruskal–Wallis test

Comparison		Age	Weight	Height	Waist	Hip	BMI	WHR
Normal pattern	Intermediate pattern	0.6843	**1.15E − 04**	**0.0078**	**6.41E − 05**	**0.0178**	**0.0138**	**0.0010**
Normal pattern	Periodic-like pattern	0.1708	**8.63E − 06**	**1.57E − 04**	**1.80E − 06**	0.1735	**0.0157**	**1.52E − 06**
Intermediate pattern	Periodic-like pattern	0.4579	0.3204	0.1969	0.2229	0.9012	0.8565	0.0604

All *p*--values highlighted in bold refer to statistically significant differences between the given breathing pattern and anthropometric parameters according to the Kruskal-Wallis test. They are less than the adopted significance level of 0.05

Groups with normal, intermediate, and periodic-like patterns of breathing did not differ according to age. Subjects with the normal breathing pattern had lower values of weight, height, waist, BMI, and WHR than persons with periodic-like and intermediate patterns. Concurrently, the two latter groups were similar regarding the abovementioned anthropometric parameters.

Additionally, there were significant differences between groups according to sex (*p* = 0.0003, chi^2^ test). It indicates that obesity (especially the so-called central obesity characterized by high WHR) and male sex were predisposing factors for the occurrence of periodic-like or intermediate patterns of respiration.

**Fig. 3:**
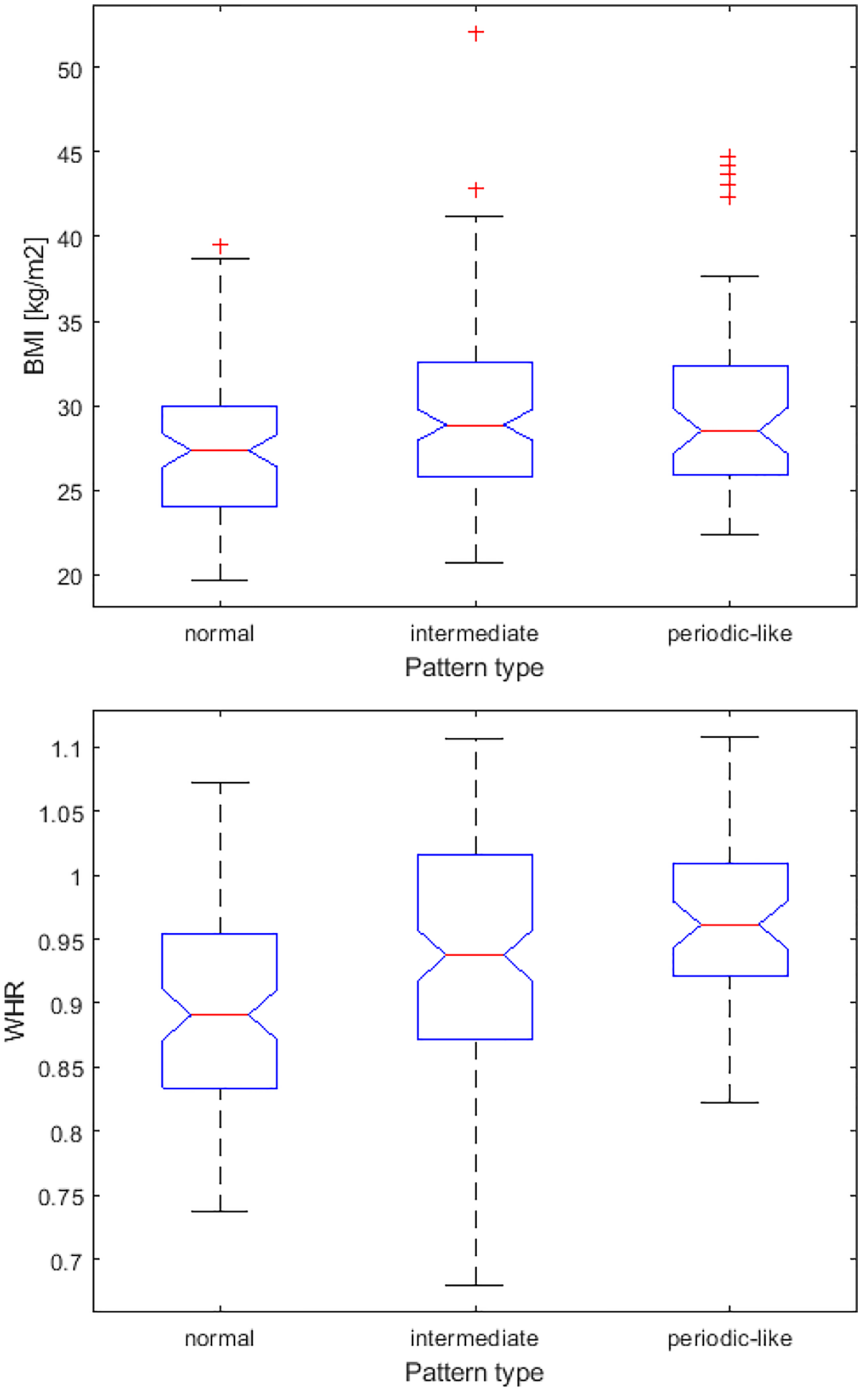
Boxplots depicting body mass index and waist-to-hip ratio values for groups with different types of breathing pattern

**Fig. 4:**
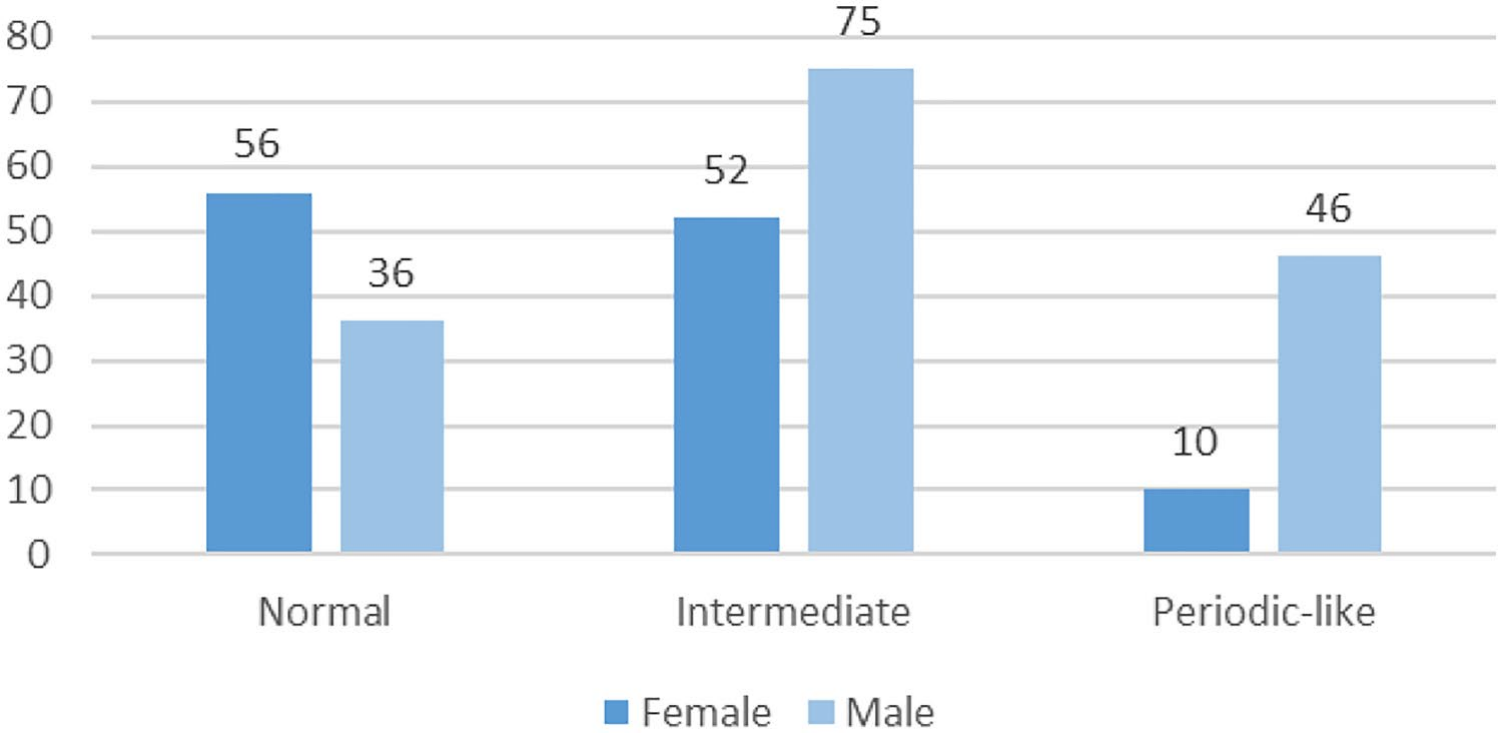
Sex structure in groups with various types of breathing pattern

The obtained results for BMI and WHR are presented in boxplots in Fig. 3. A typical data presentation was adopted; i.e., the central mark indicates the median, and the top and bottom edges of the box denote the 75th and 25th percentiles, respectively. Observations beyond the whisker length are marked as outliers using a red + symbol. Sex differences are presented in Fig. 4.

### Wavelet Analysis of Signals

The respiratory signal composition in its nature is a dynamic and non-stationary process [31], with the alternation of peaks of different spectral ranges, which is why wavelet, i.e., time-frequency representation, is useful in detecting dynamic changes of signal components and eventually observing patterns of such signal behaviors [32]. By employing scaling and translation, wavelet analysis creates a set of orthogonal basis functions. Good localization characteristics in both time and frequency domains and selectivity in the time domain make wavelet analysis suitable for approximating non-stationary signals. One of the crucial features of wavelet analysis is that it captures signal elements at different detail levels. Consequently, we call these detailed granules useful information.

#### Pre-processing of Signals

**Fig. 5:**
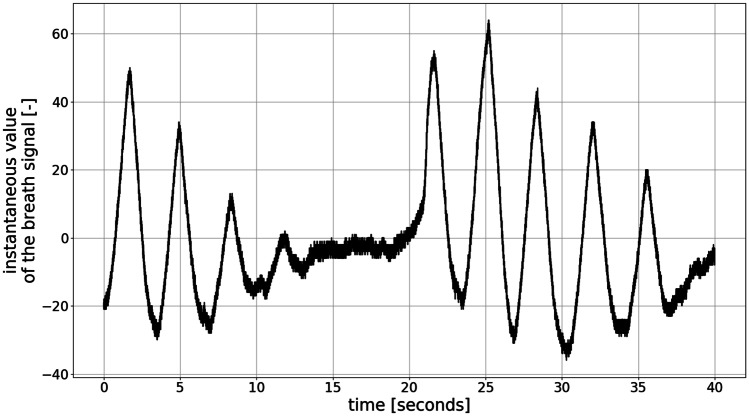
Respiratory signal obtained from a participant with a periodic-like breathing pattern. All moments of inspiration and expiration can be identified as triangular “spikes” in a signal

The analysis of the respiratory signal using machine learning methods requires performing initial pre-processing and parameterization of the input data. In our approach, these data refer to a digital signal representing changes in the participants’ chest circumferences caused by respiration. The signal has a sampling rate of 1000 Sa/s. Examples of a few seconds of such signals obtained from a healthy participant are shown in Fig. 5. Signals characterized by a “periodic-like” structure of inhale–exhale events are shown in Fig. 5.

As inspiration and expiration events may be separated by “pause” segments characterized by no significant changes in chest circumference, we have concluded that inhale–exhale events have a well-defined triangular shape of finite length, which is approximately consistent at least within a person-specific recording session. Therefore, we employed an analysis method to recognize the time of occurrence of certain inhale–exhale events in the acquired signal and frequency content analysis of those signals, namely, the analysis employing the wavelet transformation.

An important decision in designing a system for signal processing employing wavelet transformation is whether to use a continuous or discrete wavelet transformation algorithm and choose the appropriate mother wavelet for such processing. As we wanted to test some different wavelet scales, we decided to use discrete wavelet transform (DWT), which allows the decomposition of signals into components of scales, with a power of 2. Due to this property of DWT, we were able to test the scales of wavelets that had various orders of magnitude. By employing DWT, we were also able to perform calculations relatively quickly, which is another advantage of DWT compared to continuous wavelet transformation. Computation speed is important in our case, as we had to process 111.42 h of acquired respiration signals associated with participants having both normal and abnormal breathing patterns. Additionally, before calculating DWT, we first standardized the input data according to the following formula:

1 $$\mathrm{s}\mathrm{i}\mathrm{g}\mathrm{n}\mathrm{a}\mathrm{l}\mathrm{s}[\mathrm{n}]=\mathrm{s}\mathrm{i}\mathrm{g}\mathrm{n}\mathrm{a}\mathrm{l}[\mathrm{n}]-\mathrm{m}\mathrm{e}\mathrm{a}\mathrm{n}(\mathrm{s}\mathrm{i}\mathrm{g}\mathrm{n}\mathrm{a}\mathrm{l}[\mathrm{n}])\mathrm{s}\mathrm{t}\mathrm{d}(\mathrm{s}\mathrm{i}\mathrm{g}\mathrm{n}\mathrm{a}\mathrm{l}[\mathrm{n}]),$$

where

- *n* denotes a sample number.
- Signal[*n*] denotes an original, unstandardized respiration signal.
- Signals[*n*] indicates a signal after the standardization process.
- Mean() represents an operation of calculating the average of the input signal.
- Std() denotes an operation of calculating the standard deviation of the input signal.

Also, in the literature, one can find examples of DWT used for data parameterization before processing it by separate machine learning algorithms and other processing methods [33, 34], and for signal denoising [35], which also encouraged us to choose DWT as the parameterization method.

#### Choosing Appropriate Wavelet Type

**Fig. 6:**
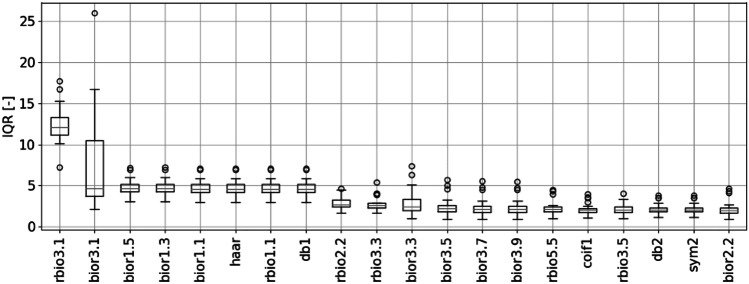
Results of IQR calculations obtained for 20 best wavelets

Another important decision regarding the pre-processing stage was the selection of the desired mother wavelet. As our signals were standardized, we assumed that scalograms generated using a better-suited mother wavelet would have a broader range of values. To perform the necessary calculations, we used the DWT by employing the PyWavelets Python library [36]. To test the wavelets from the PyWavelets library that maximized the criterion for the maximum range of scalogram values, we calculated the interquartile range (IQR) of those values that are observable in scalograms associated with each possible mother wavelet. For each of the 106 discrete mother wavelets available in the library, we calculated the IQR parameter. We averaged it over the results obtained from 30 participants classified as people having a normal breathing pattern. A trained medical doctor performed the aforementioned respiratory pattern assessment. Furthermore, we plotted the results achieved with each mother wavelet as a boxplot illustrating how the IQR values varied for every tested mother wavelet. The results of this evaluation are shown in Fig. 6. A detailed list of wavelets available in PyWavelets (we used the version 1.1.1 library) can be found in online documentation [37].

**Fig. 7:**
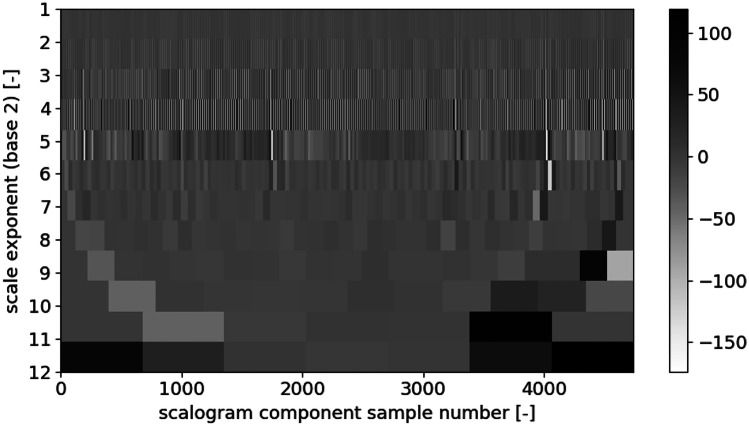
A scalogram of a participant with normal breathing pattern obtained with rbio3.1 wavelet

**Fig. 8:**
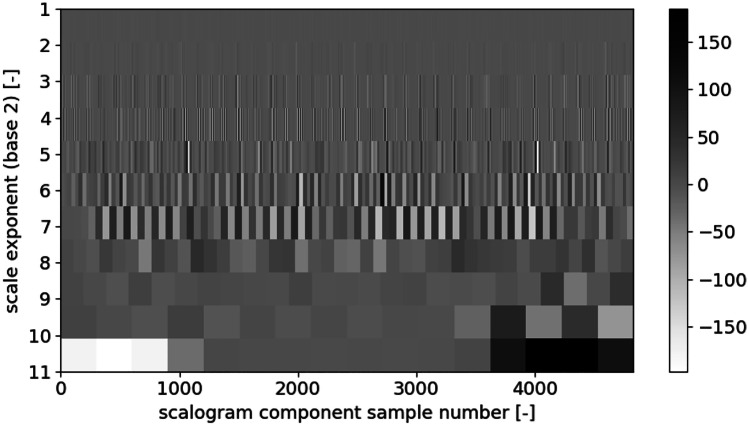
A scalogram of a participant with periodic-like breathing pattern obtained with rbio3.1 wavelet

The best mother wavelet, according to the criterion of the maximum span of the scalogram values, is rbio3.1 (Fig. 6). As DWT in the case of our analysis is intended to be the parameterization stage, we also had to reduce the input data size. To achieve this goal, we omitted seven decomposition components associated with the smallest wavelet scales. It resulted in the creation of components of approximately 4500 samples-long that is comparable to the length of a relatively high-resolution spectrogram, which machine learning algorithms can process in the next step of our analysis. Examples of final scalograms that passed the next stage of the calculations are shown in Figs. 7 and 8.

## Methods and Results

### Rough Set–Based Analysis of Data

**Fig. 9:**
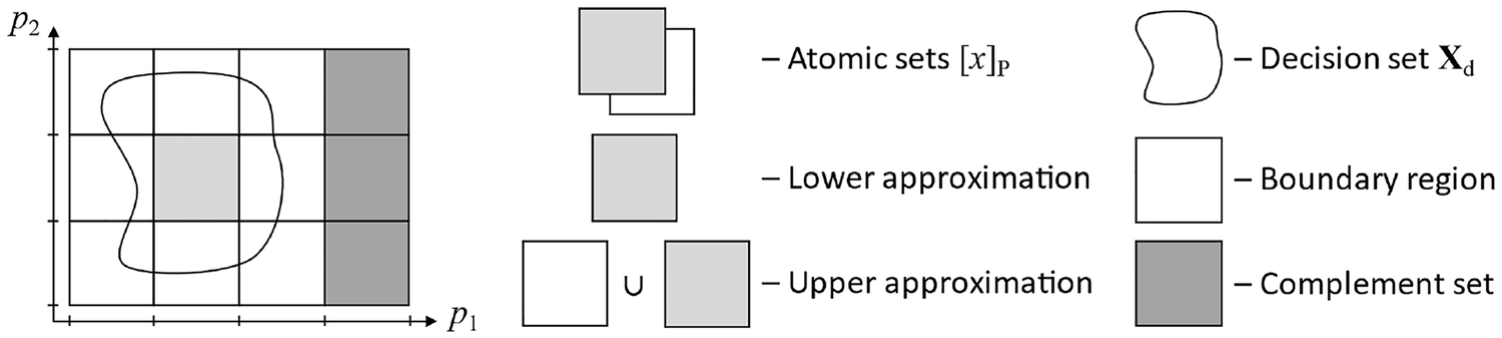
Partition of the universe based on attributes *p*_1_ and *p*_2_ into atomic sets, and approximation of the decision set **X**_d_

Rough set theory was created by Polish mathematician Zdzisław Pawlak [11, 12]. It is used to approximate a set by its upper and lower approximations: the first includes objects that may belong to the set, and the latter includes objects that surely belong to the set. Both approximations are expressed as unions of atomic sets containing indiscernible objects with the same values of attributes (Fig. 9).

Two objects *x* and *y* are characterized by attributes **P** ⊆ **A** (**P** is a subset of a set of all possible attributes **A**). These are in the indiscernibility relation if (*x*, *y*) ∈ IND(**P**), where IND(**P**) is an equivalence relation defined as a set of all pairs with exactly the same values for all considered attributes:

2 $$\mathrm I\mathrm N\mathrm D(\mathbf P)=\{(x,y)\;\in\mathbf U^2\boldsymbol\;\vert\;\forall p\in\mathbf P,p(x)=p(y)\}$$

where *p*(*x*) is the value of attribute *p* of object *x*. In this study, **P** is a set of selected wavelet types and scales introduced in the previous section, and objects *x* are particular cases of patients.

All objects in the indiscernibility relation with *x* produce an equivalence class [*x*]_**P**_, being a set of all objects identical with *x* on every attribute. If **P** contains attributes sufficient for distinguishing between objects with different decisions, then the class [*x*]_**P**_ contains only objects with the same decision as the considered object *x*. A lack of distinction between objects inside the equivalence class is not harmful to classification accuracy. Thus, P’s considered set of attributes generates a partitioning of the universe of discourse U into atomic sets, which are the building blocks for representing a rough set. A set of objects with the desired decision is such a rough set, called a decision class.

A set of all objects with one of the possible decisions *d* = {*d*_1_,…, *d*_n_} is denoted as **X**_di_. Following the rough set theory, **X**_di_ can be approximated by its lower and upper approximations, the former denoted as $\underline{\mathbf P}{\mathbf X}_{\mathrm d\mathrm i}$:

3 $$\underline{\mathbf P}{\mathbf X}_{\mathrm d\mathrm i}=\{x\;\vert\;{\lbrack x\rbrack}_{\mathbf P}\subseteq{\mathbf X}_{\mathrm d\mathrm i}\}$$

The lower approximation is a set of all objects *x*, whose equivalence classes [*x*]_**P**_ are included within the decision class of interest **X**_di_. It can also be interpreted as a set of objects whose attribute values allow for precise classification of the decision class **X**_di_ with a decision *d* = *d*_i_.

Also, the set of objects $\overline{\boldsymbol{P}}$**X**_di_ is called upper approximation and is defined as:

4 $$\overline{\boldsymbol P}{\mathbf X}_{\mathrm d\mathrm i}=\left\{x\;\vert\;\left({\left[x\right]}_{\mathbf P}\cap{\mathbf X}_{\mathrm d\mathrm i}\right)\neq\varnothing\right\}$$

The upper approximation includes all objects *x*, whose equivalence classes have a non-empty intersection with the considered decision class **X**_di_. It can be conveniently interpreted as a set of all objects *x* that have the values of their attributes pointing to similar objects, and at least one of these has the desired decision **X**_di_. Some object(s) equivalent to *x* can have other decisions as well (Fig. 9).

The given subset of attributes **P** can be sufficient enough to generate such a partitioning of the universe of *x* ∈ **U** that decision classes are approximated with high precision. The accuracy of the rough set approximation of a decision class **X**_di_ is expressed as

5 $${\alpha }_{P}\left({\mathbf{X}}_{\mathrm{d}\mathrm{i}}\right)=\frac{\left|\underline{\boldsymbol{P}}{\mathbf{X}}_{\mathrm{d}\mathrm{i}}\right|}{\left|\overline{\boldsymbol{P}}{\mathbf{X}}_{\mathrm{d}\mathrm{i}}\right|}$$

and ${\alpha }_{P}\left({\mathbf{X}}_{\mathrm{d}\mathrm{i}}\right)$ ∈ [0,1], where ${\alpha }_{P}\left({\mathbf{X}}_{\mathrm{d}\mathrm{i}}\right)$= 1 will be the case of a precisely defined crisp set.

Application of the rough set theory in a decision system often requires a minimal (the shortest) subset of attributes **RED** ⊆ **P**, called reduct, resulting in the same quality of approximation as **P**. Numerous algorithms for calculating reducts are available, and for this study, two methods are examined [38] (described in the “Experimental Procedure” section).

Usually, prior to reduct calculation for attributes with continuous values, discretization is performed. Discretization algorithm analyses attribute domain, sorts values present in the training set, takes all midpoints between values, and finally returns the midpoint maximizing the number of correctly separated objects of different classes. It is repeated for every attribute. Three different methods were examined in this study. Discretization limits the number of possible values—for attributes in this study, there are 1, 2, or 3 cuts, splitting the values into 2, 3, or 4 discrete ranges, accordingly.

Once the reduct is obtained, the attributes useful for a particular classification task are known. The data are filtered, the attributes not present in the reduct are removed, and others are discretized accordingly. Furthermore, all cases in the training set are analyzed, and decision rules are generated. Each object *x*_i_ attributes *p*_n_ ∈ **RED** are treated as an implication antecedent, and the decision *d*_i_ for the *x*_i_ object is the rule consequent. Rules in the form of logical sentences are obtained:

6 $$\mathrm I\mathrm F\;p_1\left(x_{\mathrm i}\right)=v_1\;\mathrm A\mathrm N\mathrm D\;\dots\;\mathrm A\mathrm N\mathrm D\;p_{\mathrm n}\left(x_{\mathrm i}\right)=v_{\mathrm n}\;\mathrm T\mathrm H\mathrm E\mathrm N\;d\left(x_{\mathrm i}\right)=d_{\mathrm i}$$

At the classification phase, these rules are applied for every object in the testing set, and subsequently, the decision is determined to be compared with the actual one and measure the accuracy.

The abovementioned treatment is initialized 10 times in a 10-fold cross-validation procedure, each time comprising determining the discretization cuts, the reduct, and generating the rules based on the training set, then applying the rules to classify the testing set, and measuring the accuracy. The process is automated by employing a script written in the R language [39].

#### Other Rough Set Approaches

The process described above assumes a crisp distinction between atomic sets. A rough set theory variant called fuzzy rough sets applies fuzzy equivalence classes, making use of fuzzy indiscernibility [40, 41]. It allows for expressing imprecise knowledge about similarity and dissimilarity between objects by fuzzy membership functions. The presented study uses crisp atomic sets as a result of cut calculation; therefore, the fuzzy approach is unsuitable here.

Another interesting extension of rough sets is the dominance-based approach. It requires all attributes to follow some preference order, where it is possible to determine the more and the less desired values [42]. In this study, the features extracted by wavelet analysis cannot be ordered by preference; therefore, this approach is not applicable.

#### Dataset Description

Decision features of periodic-like, *irregular*, and *correct* breathing patterns contain percentage measures determined by a medical expert, expressing how strongly a given pattern type is present in the signal (for simplification of records and presentation of results, they are converted to deciles 0, 1, 2, …, 10).

There are significantly more cases with 0 values than others; therefore, a stratification—the bias reduction procedure—is introduced by purposefully sampling the dataset to obtain as many cases with 0 values as the average number of cases with values 1, 2, ...,10.

##### Goal

Therefore, the goal was to explore a rough set–based [11, 12] highly granularized approach for data mining of a breathing pattern database. It was shown here how signal attributes and anthropomorphic parameters could be exploited to create prediction models to determine the percentage contribution of periodic-like, intermediate, and normal breathing patterns in the analyzed signals. The output class values are quantized, and many possible quantization ranges are verified during the automatic search of optimal model hyperparameters aimed at maximizing the resulting model accuracy. As already mentioned, the R programming environment [39] with the RoughSets package [38, 43−45] was used for the rough set–based processing.

The model hyperparameters considered in this study are as follows: (1) the number of quantization cuts for the input data discretization and (2) the ranges defining output classes that had been provided with a 10-value scale but were quantized into three ranges in the process.

##### Data Granularization

For knowledge extraction and modeling, two initial assumptions were made:

- The input signal wavelet parameters that have continuous values and varying lengths, dependent on the signal length and wavelet scale, were quantized for the processing and reduced to a fixed number of only four descriptors for each wavelet scale, based on quartile ranges.
- The output classes with 10 values of percentage contribution are quantized into three ranges: low, medium, and high (coded as “A,” “B,” “C”), defined by discretization cuts.

Consequently, a separate fine-tuned model was created for each breathing pattern that outputs one label (low, medium, and high) easily for interpretation. These classifiers are suitable for operation on input signals of any length.

##### Discretization of Wavelet Parameters

Only three wavelet scales were selected: 5, 6, and 7, which were the compromise between matching the time resolution to a breathing period and choosing some samples that would be low enough to assure fast processing. It may be observed in Figs. 7 and 8 that scales higher than 7 are too coarse and do not exhibit the breathing cycles inherent periodicity. Scales lower than 5 are too detailed and appear to contain the same information as 5, 6, and 7.

From each wavelet scale, the following four descriptors are extracted: first, second, and third quartiles (Q1, Q2, and Q3) and interquartile range (IQR = Q3–Q1), serving as a summary of the signals regardless of their lengths and disregarding the various wavelet scales (Table 3).

**Table 3:** Sample values of descriptors calculated for two different breathing patterns (high IQR values can be observed for periodic patterns)

Sample descriptors of a normal pattern				Sample descriptors of a periodic-like pattern			
	Scale 5	Scale 6	Scale 7		Scale 5	Scale 6	Scale 7
Q1	−2.54	−6.70	−6.06	Q1	−11.17	−19.93	−50.13
Q2	−0.21	0.00	1.20	Q2	4.51	13.05	13.69
Q3	12.15	5.84	5.01	Q3	15.38	30.95	53.19
IQR	24.70	12.55	11.07	IQR	26.56	50.88	103.33

Therefore, the general signal characteristics are obtained, are robust to noise, and are suitable for further processing in a rule-based decision system. Those real-valued descriptors are further quantized during the rough set–based knowledge modeling process, employing one of the selected discretization algorithms (global discernibility, unsupervised intervals, or unsupervised quantiles), with a set number of cuts *c*. The discretization method and *c* were considered as arguments for exploration during the automatic search for model hyperparameters.

##### Discretization of Output Classes

The output was initially defined on a 10-valued decile scale, describing the intensity of a particular breathing pattern in the analyzed recording. For this study, it was transformed into only three ranges, automatically during the model search, by setting a set D = {d, d2}, where d1 = {1, 2, 3, 4, 5} is a lower decile boundary, and d2 = d1 + width is a higher decile boundary, where width = {2, 3, 4, 5}, with a condition d2 < 9 fulfilled. During the discretization, the pair of cuts D = {d, d2} were implemented as ranges [0, d1), [d1, d2), [d2, 10), and replaced with labels “A,” “B,” and “C,” respectively. The structure of the resulting decision table exploited in the following experiments is presented in Table 4.

**Table 4:** The structure of the discretized decision table

Scale5_q1	Scale5_q2	Scale5_q3	Scale5_IQR	Scale6_q1	Scale6_q2	Scale6_q3	Scale6_IQR	Scale7_q1	Scale7_q2	Scale7_q3	Scale7_IQR	Age	Sex	BMI	WHR	Contribution of the pattern
																A
																B
																C

### Experimental Procedure

Each experimental run comprises several key steps, including selecting the modeled pattern, data filtration for bias reduction, splitting into training and testing cases (in a ratio of 85:15), and training the model with given hyperparameters, verifying the accuracy. For each combination of hyperparameters, 10 such runs were conducted, each with different random data filtration and splits, acting as a cross-validation method and coping with a relatively low number of cases in the database. A detailed description of the procedure is as follows:

- From normal, intermediate, and periodic-like, choose the breathing pattern to be modeled:
  - ◦ Set the hyperparameters of the model to be trained.
  - ◦ Set the number of attribute value discretization cuts c = {1, 2, 3}.
  - ◦ Set the discretization method (global discernibility, unsupervised intervals, or unsupervised quantiles).
  - ◦ Set the reduct computation method (DAAR heuristic or greedy heuristic).
  - ◦ Set the rule induction methods (LEM2, CN2, AQ, or rule induction from indiscernibility classes).
  - ◦ Set the output class discretization ranges D = {d1, d2}.
- Filter the data: Reduce the risk of bias by randomly subsampling the cases where the decile value is equal to 0.
  - ◦ Calculate the average number of cases where the decile value is equal to 1, 2, …, 10.
  - ◦ Count all cases where the decile value is equal to 0.
  - ◦ Calculate how many 0 cases should be removed to match their number to the average.
- Divide randomly into training and testing sets in 85:15 ratios.
- Apply one of the selected discretization methods for attribute values, using the c cuts.
- Calculate the reduct and rules.
- Apply the rules to the test cases.
- Measure and report the accuracy of the results and the number of rules.
- Repeat 10 times for the same hyperparameters, and create statistics for accuracies and numbers of rules.

To summarize, the whole process explores hyperparameters: c, discretize method, reduct algorithm, rule algorithm, decision ranges d1 and d2, to automatically find the model configuration that maximizes resulting prediction accuracy for the decision classes of the breathing pattern. It can be formalized as follows:

7 $$\underset{\{c,discretise,rule,reduct,d1,d2\}}{\mathrm{a}\mathrm{r}\mathrm{g}\mathrm{m}\mathrm{a}\mathrm{x}}mean(accu\left(model\left({X}_{c,discretise},rule,reduct\right),{decision}_{d1,d2}\right))$$

#### Model Exploration Results

**Fig. 10:**
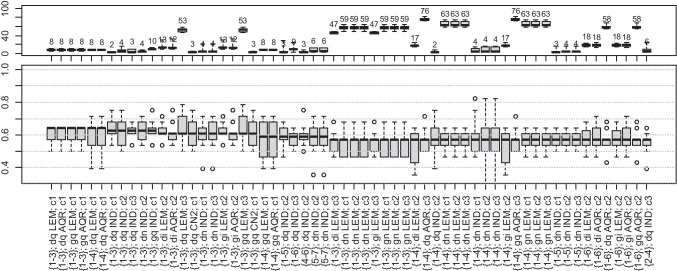
Length of rules (top, median lengths are shown above the boxplots) and accuracy (bottom) for classification of periodic-like cases to target decile ranges (sorted by decreasing median of accuracy). The legend is contained in the text under the “Model Exploration Results” section

**Fig. 11:**
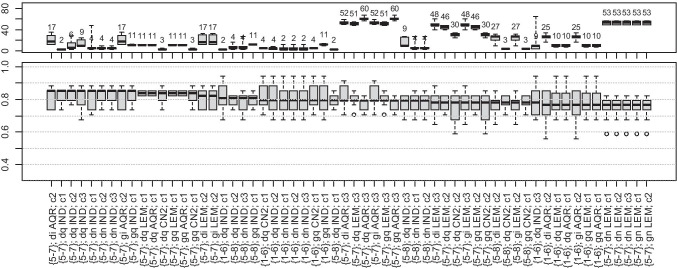
Length of rules (top) and accuracy (bottom) for classification of intermediate pattern cases to target decile ranges (sorted by decreasing median of accuracy). The legend is contained in the text under the “Model Exploration Results” section

**Fig. 12:**
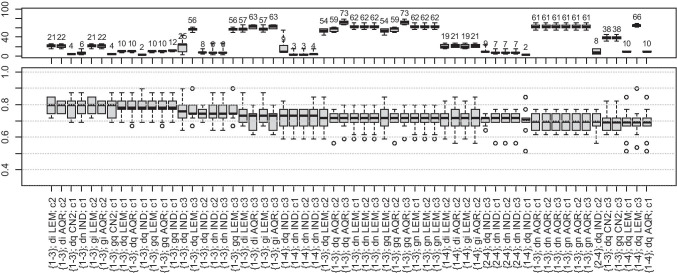
Length of rules (top) and accuracy (bottom) for classification of normal pattern cases to target decile ranges (sorted by decreasing median of accuracy). The legend is contained in the text under the “Model Exploration Results” section

The results of 60 best model exploration runs are presented in Figs. 10, 11, and 12, sorted by decreasing median accuracy. Accuracies and the number of rules in the generated models were collected and presented as boxplots (with quartiles and medians). On the *x*-axis, the labels denote the model configuration, where

- {d1–d3}, e.g., {1–3}, is a definition of modeled decile range cuts.
- *d* or *g* is an abbreviation for reduct computation method: DAAR heuristic, or greedy heuristic.
- *q*, *i*, or *n* is an abbreviation for the discretization method: unsupervised quantiles, unsupervised intervals, or global discernibility.
- LEM2, CN2, AQ, and IND are rule-induction methods.
- *cn* is the number of desired cuts for attribute discretization.

#### Accuracy and Rule Analysis

A process of rule filtration was performed based on a few criteria. First, models with less than two rules were removed because to perform a decision regarding three classes, at least two rules should be employed (for example, if the 1st rule is true—the class can be “A”; if the 2nd rule is true—the class can be “B”; and if neither is true—the class can be “C”). Then, models with a mean Laplace confidence calculated over all the model rules less than 0.6 were removed. Laplace confidence is a metric reflecting rule accuracy over the considered class and all objects matching the rule:

8 $$Lc({R}_{K})=\frac{{n}_{K}({R}_{K})+1}{n({R}_{K})+k}$$

where ${R}_{K}$ is the rule related to the class *K*, ${n}_{K}({R}_{K})$ is the number of objects of the class *K* correctly classified by the rule, $n({R}_{K})$ is the number of all objects matching the rule (regardless of their class), and *k* is the number of classes in the model.

If the number of rules with Lc > 0.6 was larger than 100, the threshold was decreased to leave at most 100 rules in the rule base for each examined model. It was assumed that such a high number of rules for classification into three classes is excessive and impractical.

Notably, other rule selection methods were examined as well: support and confidence, and the results were confirmed to be similar (the result accuracies reported in this section are similar with a significance of 0.05).

It can be observed from Table 5 that the percentage of contributions of the periodic-like pattern is the most problematic relation to the model, and no approach resulted in an accuracy higher than 0.65 (Fig. 10). Then, the relationship between signal attributes, anthropometric features, and the resulting percentage of intermediate and normal patterns is more clearly defined, and median accuracies in a considerable number of cases exceed 0.75 (Figs. 11 and 12).

**Table 5:** Average accuracies for breathing patterns and decision discretization ranges: d1, d2

Periodic-like								Intermediate								Normal							
		d2								d2								d2					
		3	4	5	6	7	8			3	4	5	6	7	8			3	4	5	6	7	8
d1	1	0.79	0.82	0.68	0.79			d1	1	0.65	0.74	0.85	0.94			d1	1	0.9	0.92	0.85	0.8		
	2		0.82	0.68	0.71	0.68			2		0.65	0.68	0.71	0.73			2		0.8	0.8	0.77	0.64	
	3			0.71	0.68	0.68	0.71		3			0.71	0.68	0.71	0.74		3			0.77	0.72	0.74	0.77
	4				0.75	0.75	0.71		4				0.83	0.79	0.79		4				0.77	0.72	0.77
	5					0.75	0.71		5					0.91	0.85		5					0.74	0.72

Table 6 reports the average F1 scores. It can be observed that values fall below 0.74 for the considered problem of three classes with imbalanced sizes, which is a very common problem. The best cases are as follows: for the periodic type for D = {d1, d2} = {3, 8}, when the average accuracy is 0.71, the average precision is 0.72, the average recall is 0.75, and average F1 = 0.72; for the intermediate type for D = {d1, d2} = {1,6}, when the average accuracy is 0.94, the average precision is 0.71, the average recall is 0.8, and average F1 = 0.72; and for the normal type for D = {d1, d2} = {4,8}, when the average accuracy is 0.77, the average precision is 0.77, the average recall is 0.73, and average F1 = 0.74. Therefore, in these cases, a dedicated classifier based on considered models can be implemented and used in practice for a screening procedure and automatic coarse determination of the breathing pattern.show that a considerable

**Table 6:** Average F1 scores for breathing patterns and decision discretization ranges: d1, d2

Periodic-like								Intermediate								Normal							
		d2								d2								d2					
		3	4	5	6	7	8			3	4	5	6	7	8			3	4	5	6	7	8
d1	1	0.54	0.58	0.62	0.59			d1	1	0.55	0.42	0.45	0.72			d1	1	0.60	0.64	0.50	0.51		
	2		0.59	0.60	0.52	0.44			2		0.55	0.43	0.57	0.59			2		0.58	0.54	0.72	0.60	
	3			0.49	0.64	0.54	0.72		3			0.65	0.40	0.47	0.43		3			0.63	0.48	0.71	0.72
	4				0.43	0.61	0.53		4				0.50	0.64	0.46		4				0.41	0.62	0.74
	5					0.54	0.71		5					0.63	0.47		5					0.54	0.67

#### Rule Analysis

All signal attributes (wavelet scales and quartile ranges) were present in the models. In very few cases, the rules incorporated anthropomorphic features, namely age, and WHR.

Figures 10, 11, and 12 show that a considerable number of possible approaches can result in similar classification accuracy, and some of the tested decile ranges produce higher results than others (Table 5). Regardless of attempts to reduce bias in the dataset, the procedure remained flawed in this regard. In many models, the extracted knowledge is oriented towards a single class with high accuracy instead of all. Notably, many rules describe only one class, the one with the highest number of cases, resulting in low overall accuracy. It occurs for wide decile ranges, i.e., when one range covers a significantly larger number of cases than any other range. Then, the model tends to favor this particular class, deriving more rules supporting these cases.

##### Non-biased Rules

For d1 = 3 and d2 = 6, the results are not biased, as the resulting discretization ranges contain an approximately equal number of cases and have a similar width.

An example of a model comprising four rules describing the percentage of the normal pattern contribution is as follows (number of training cases supporting the rule is provided in the brackets at the end of each rule):

1. IF scale5_q2 is [0.921, Inf] and scale7_iqr is [18.1, Inf] THEN *Normal* is A (72).
2. IF scale5_q2 is [− Inf, 0.921) and scale7_iqr is [18.1, Inf] THEN *Normal* is B (38).
3. IF scale5_q2 is [− Inf, 0.921) and scale7_iqr is [− Inf, 18.1) THEN *Normal* is C (71).
4. IF scale5_q2 is [0.921, Inf] and scale7_iqr is [− Inf, 18.1) THEN *Normal* is C (38).

The resulting accuracy is 0.72. The above rule set is consistent (no two rules contradict each other) and can be used in further experiments.

##### Incomplete Rule Set

The same target ranges (d1 = 3, d2 = 6) for periodic type produced only two rules:

1. IF scale7_q1 is [−9.69, Inf] THEN Periodic is A (79).
2. IF scale7_q1 is [− Inf, −9.69) THEN Periodic is C (79).

The classification accuracy for the test cases is 0.75. It can be observed that such a model does not detect range B of the periodic pattern contribution, and it tends to make a bimodal decision, either to class A (contribution less than 30%, as the decile margin is d1 = 3) or to class C (contribution higher than 60%, decile d2 = 6).

##### Contradicting Rules

Example rules for determining the percentage contribution of the normal pattern to the centile ranges defined by d1 = 1 and d2 = 3 are as follows:

1. IF scale5_q2 is [−3.82,13.1) and scale6_q2 is [−3.37,9.08) THEN *Norma*l is C.
2. IF scale5_q2 is [−3.82,13.1) THEN *Normal* is A.
3. IF scale5_q1 is [−41, −2.22) THEN *Normal* is B.

The above rules were derived by applying DAAR heuristics for reduct calculation, unsupervised quantiles for discretization, and the CN2 algorithm for rule generation.

It can be observed that rule no. 2 is in contradiction with rule no. 1, but during the inference, it is interpreted that all cases matching the more specific rule no. 1 (two attributes checked in the antecedent) are classified as a class C (the contribution of normal pattern in centile d2 = 3 or higher). Then, cases not covered by rule no. 1 but are covered by rule no. 2 are classified as A. The accuracy here is 0.795 and can be considered appropriate for screening applications.

### Data Analysis Based on the k-Nearest Neighbor Algorithm

Discretized data used for training of the rough set–based system were also subject to the analysis employing a relatively simple classification method—the k-nearest neighbor algorithm. It can be a baseline approach that is useful in assessing the robustness of a solution employing rough sets. Despite being simple in principle, the k-nearest neighbor algorithm also has some hyperparameters that can be optimized. We used a grid search approach to select the best values for

- Discretization level $$d$$.
- Normalization method of data after discretization.
- Number of neighboring points considered in the classification process, which is denoted as $k$.
- Type of Minkowski distance metric is used for finding the nearest neighbors.

For the distance metric, the Minkowski distance was employed. It is a convenient choice for optimization, as the type of Minkowski distance can be controlled with a parameter, as the formula for calculating such a metric is as follows:

9 $${D}_{Minkowski}\left(\boldsymbol{x},\boldsymbol{y}\right)={\left(\sum _{i=1}^{n}{\left|{x}_{i}-{y}_{i}\right|}^{p}\right)}^{\frac{1}{p}},$$

where ${D}_{Minkowski}\left(\boldsymbol{x},\boldsymbol{y}\right)$ denotes the Minkowski distance between points $\boldsymbol{x}$ and $\boldsymbol{y}$, $n$ is the number of dimensions, and $p$ is a parameter that can be optimized in the grid search procedure.

The grid search was conducted for all discretization ranges considered in the rough set–based experiment. For data normalization, five possibilities were considered:

1. No normalization.
2. Normalization by division of parameters (which can also be called dimensions, columns of the dataset) by the maximum absolute value of such parameter.
3. Normalization by scaling parameters to the range of $<-1;1>$.
4. Standardization, which is performed by performing calculations according to the following formula:
  10 $${x}_{standardized}=\frac{\boldsymbol{x}-\overline{x}}{\mathrm{s}\mathrm{t}\mathrm{d}\left(\boldsymbol{x}\right)},$$
  where ${x}_{standardized}$ is a dimension after normalization, $\boldsymbol{x}$ is a vector before normalization, $\overline{x}$ is the mean value of $\boldsymbol{x}$, and $\mathrm{s}\mathrm{t}\mathrm{d}\left(\boldsymbol{x}\right)$ is a standard deviation of $\boldsymbol{x}$.
5. Normalization of data points (which correspond to rows of the dataset) by treating them as vectors and normalizing the length of such vectors to 1, which can be defined as follows:
  11 $${\boldsymbol{u}}_{\boldsymbol{d}.\boldsymbol{p}.\boldsymbol{n}\boldsymbol{o}\boldsymbol{r}\boldsymbol{m}.}=\frac{\boldsymbol{u}}{\left|\boldsymbol{u}\right|},$$
  where ${\boldsymbol{u}}_{\boldsymbol{d}.\boldsymbol{p}.\boldsymbol{n}\boldsymbol{o}\boldsymbol{r}\boldsymbol{m}.}$ is a vector representing given data point after normalization, $\boldsymbol{u}$ is a vector representing data point before the normalization, and $\left|\boldsymbol{u}\right|$ is the length of the aforementioned vector.

Notably, the last type of normalization is applied to data points, not parameters themselves. For the number of neighbors ($k$), values from 2 to 50 were considered. For the Minkowski distance, there were five values of $p$ used, namely 1 (for which the metric becomes a so-called Manhattan distance), 1.25, 1.5, 1.75, and 2 (for which the metric becomes the Euclidean distance). The results obtained in a grid search are shown in Table 7. Overall, 20,825 combinations of hyperparameters were evaluated. For the evaluation of the performance, an approach based on twofold cross-validation repeated five times (5 × 2CV) was employed [46]. As classes for each discretization range were not balanced, only the F1 score was used for evaluation of the performance. A resulting performance estimate in the form of descriptive statistic parameters was provided. The aforementioned statistical parameters are minimum and maximum values of the F1 score, the mean F1 score, its standard deviation, and the confidence interval for the F1 score with the 95% significance level. Gaussian distribution of the performance metric was assumed and used in the calculation of the confidence intervals. Implementations of k-nearest neighbors and cross-validation are from the scikit-learn Python library (version 0.24.1). For all parameters not specified in this study, a default value was used.

**Table 7:** A ranking list of 20 best-performing sets of hyperparameters (regarding the largest F1 score value obtained) used to perform the 5 × 2CV procedure using the k-nearest neighbor classifier. Sets of hyperparameters are shown on the left side of the table, and statistical measures describing the level and variation of performance measured by the F1 score are shown in the right part of the table

Pre-processing type and hyperparameters				Achieved F1 score statistics				
d	Normalization type	k	$p$	Min	Mean	Max	Std. dev	Conf. int. (95%)
{4,8}	None	41	1.5	0.557	0.617	0.676	0.059	(0.500; 0.733)
{4,8}	None	41	1.75	0.526	0.590	0.654	0.064	(0.464; 0.716)
{4,8}	None	28	1.25	0.569	0.609	0.648	0.040	(0.531; 0.687)
{4,8}	None	41	1.25	0.560	0.602	0.645	0.043	(0.519; 0.686)
{4,8}	None	41	2	0.509	0.575	0.641	0.066	(0.446; 0.704)
{4,8}	Standardization	28	1	0.598	0.617	0.637	0.020	(0.579; 0.656)
{4,8}	None	41	1	0.594	0.615	0.636	0.021	(0.574; 0.656)
{2,6}	None	16	2	0.562	0.598	0.635	0.036	(0.528; 0.669)
{4,8}	Standardization	28	1.25	0.605	0.617	0.630	0.012	(0.593; 0.642)
{4,8}	Standardization	41	1.75	0.570	0.600	0.629	0.029	(0.542; 0.657)
{3,8}	None	16	1.25	0.602	0.615	0.628	0.013	(0.589; 0.640)
{4,8}	Standardization	41	1.5	0.556	0.589	0.622	0.033	(0.524; 0.654)
{3,8}	Standardization	16	1.75	0.539	0.580	0.622	0.041	(0.499; 0.661)
{4,8}	None	28	1.5	0.583	0.602	0.621	0.019	(0.565; 0.640)
{3,8}	None	28	1.25	0.578	0.599	0.619	0.020	(0.559; 0.639)
{3,8}	None	16	1	0.597	0.608	0.619	0.011	(0.587; 0.630)
{4,8}	Standardization	28	1.5	0.572	0.595	0.617	0.022	(0.551; 0.638)
{4,8}	None	28	1.75	0.568	0.592	0.616	0.024	(0.545; 0.639)
{3,8}	Standardization	16	1	0.559	0.587	0.615	0.028	(0.532; 0.642)
{2,7}	None	16	1.5	0.560	0.587	0.615	0.028	(0.533; 0.641)

In Table 7, one can find that the most commonly occurring type of discretization is {4,8} and the maximum obtained F1 score is 0.676, which is a worse result than the one obtained by the rough set–based approach. However, it also should be stressed that the higher boundary of the confidence interval is just slightly lower than the accuracy of the rough set–based approach, which is equal to 0.74. Also, it is clear that in most cases, it is beneficial to not use the Euclidean distance in measuring distances and choose values of $p$ between 1 and 2. The most common values of $p$ are 1.75 and 1.5. Both of them occurred five times throughout the ranking list. From all investigated normalization types, only standardization was found to be present among the 20 best sets of hyperparameters.

**Fig. 13:**
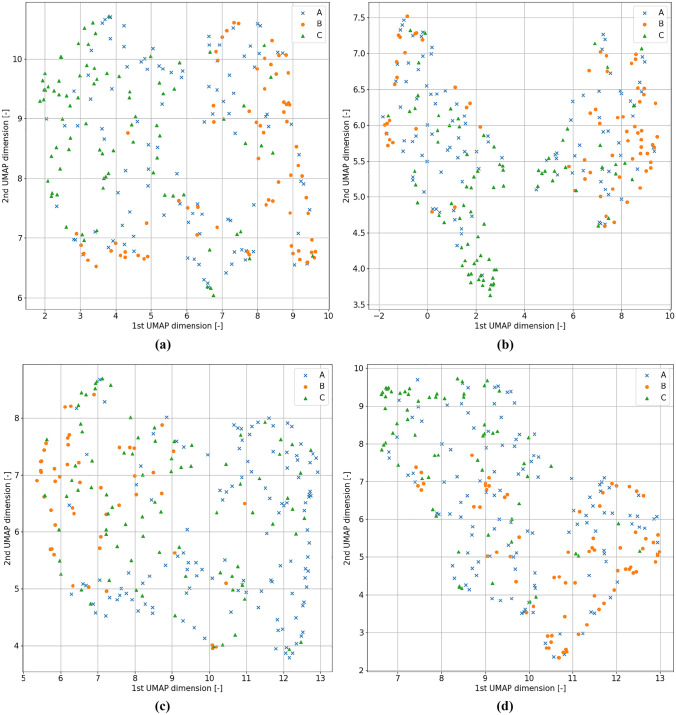
Visualizations of the points in the decision space generated with the UMAP algorithm. For **a**, the scenario of {4,8} discretization range and no data normalization is shown. For **b**, there was a {4,8} discretization range and standardization was employed as the normalization method. For **c**, a {2,6} discretization range was used with no normalization. Lastly, for **d**, a {3,8} discretization range with no normalization was used

To visualize the structure of the data classified by the k-nearest neighbor algorithm, a visualization was prepared for the four most effective discretization ranges and pre-processing types. The results of such visualization prepared by employing the UMAP dimensionality reduction algorithms are shown in Fig. 13 [47]. An implementation of UMAP available in the umap-learn Python library (version 0.5.1) was used.

All the visualizations presented in Fig. 13 show clusters having the property that different classes tend to be most prominent in just one specific region of the cluster. However, despite scenario b where standardization was used, there are no separate clusters present. In scenario b, those clusters are likeliest to correspond to two sexes of participants and have no evident association with classes A, B, or C. This implies that even for the data pre-processing types, which were the most beneficial for the k-nearest neighbors, there was no obvious way to separate examples of at least some classes. There is always some overlap between them. However, one can identify situations, such as one visible in the subplot c, where classes A and B are separated by a significant amount of margin for most of the data points belonging to one of them.

## Discussion

The accuracy and F1 scores obtained by the tri-way reasoning employing rough set–based approach was relatively high and amounted to 0.795 (accuracy) and 0.74 (F1 score), respectively, so it already aided respiratory pattern evaluation. Yet, implementing the rule set is not straightforward, and further expansion with new knowledge can be problematic. The approach in modeling various percentage contribution ranges should be further explored to reduce the bias and achieve possible higher accuracy for narrow centile ranges. It was shown that for evenly spread ranges <0,3>, <3,6>, <6,10> defined by d1 = 3, d2 = 6 results in lower accuracy but more appropriate rule sets (complete rule sets without bias). Therefore, this issue will be studied in the future. When comparing the results of the rough set–based analysis with a baseline algorithm (k-NN), it occurs that rough sets return higher values. On the other hand, employing k-NN and UMAP visualization brought new insights into the data gathered. The UMAP-based approach shows that different classes tend to be most prominent in just one specific region of the decision space. However, despite various standardization scenarios used, there are no separate clusters present.

Moreover, the database will be extended with new cases, allowing the research to focus on a lower number of models but potentially contain more accurate and usable knowledge, automatically extracted from a larger number of training samples.

Our study indicates that the granularity concept applied to respiratory rate quantification and abnormal pattern prediction might provide novel insights into cardiorespiratory regulation beyond those offered by a simple analysis of respiratory rate, inspiration and expiration times, tidal volume assessment, or their variability [48, 49]. Many previous studies concerning respiratory variability have been performed in animals and cannot be directly translated to humans [50, 51]. Analysis of the pattern of respiratory signals in humans is much more challenging. The variability of frequency and amplitude, the impact of artifacts (related to body movement, speech, etc.), and the individuality of patterns in different subjects should be considered [52]. Traditionally, visual assessment by an expert physician has often been used to identify PB appearance. However, this approach is mainly subjective and might be misleading. Previous efforts to develop better methods were either limited to the investigation of respiratory patterns during sleep from polysomnographic recordings [14] or performed in homogeneous groups of subjects: healthy individuals [22, 53], neonates [26], or patients with CHF [16, 54]. Furthermore, these studies aimed to detect patients with clear-cut periodic patterns of breathing.

## Conclusion

This study was conceived as a tri-way approach to evaluate breathing patterns automatically. We started by observing how a medical expert performs measurement, collects the data and signals, and evaluates and interprets them to form knowledge. Followed by that, group reasoning was executed by physicians, diagnosticians, and computer scientists. Collectively, a sequence of tasks was envisioned concerning the methodology regarding structuring the collected data, deciding on the signal analysis, and the processing method. This stage outcome used wavelet-based signal analysis and data and signal processing by rough sets. We incorporated these data processed into granules representing knowledge related to a particular patient. An important decision was to select an appropriate type of wavelet analysis, i.e., continuous (CWT) or discrete (DWT). The outcome of the discussion related to this end was the use of DWT.

Furthermore, we formulated a criterion upon which the desired mother wavelet was chosen. Concerning the results obtained, interestingly, all signal attributes were present in the models. Contrarily, the rules rarely incorporated anthropomorphic features. It should be further explored since some of these data are regarded by a medical expert as substantial. However, obesity (especially the so-called central obesity characterized by high WHR) and male sex were predisposing factors for the occurrence of periodic-like or intermediate patterns of respiration. It may be one of the essential findings derived from this study. Even though BMI is used as a primary assessment tool in numerous fields in medicine linked to poor health outcomes, WHR, waist (circumference)-to-hip (circumference) ratio, may be a better indicator related to faulty breathing patterns, as it considers different body types, sex, and age. This finding confirms the work by Ross et al. who posited that WHR is a more critical factor than BMI in medical assessment [55].

To reassume, in this study, we considered three patterns of breathing—normal, intermediate, and periodic-like—in a group of subjects of different ages, sex, body constitution, and medical records (healthy, hypertensives, patients with a history of TIA, etc.). It is an important distinction that our approach, based on wavelet analysis along with rough set–based processing, was effective in non-invasive and short-term (20-min) recordings during wakefulness. Analyses performed by employing k-NN validated to some extent the results obtained in the rough set–based approach, though the obtained F1 score values were smaller. Also, it seems that the UMAP visualization allows for evaluating the data gathered, showing that clusters tend to form themselves in various parts of the decision space; however, they are not fully separated.

Our results indicate that the proposed method can support the visual assessment of respiratory patterns by an expert. Automatic characterization of breathing patterns, based on our approach, can be applied in future studies focusing on cardiorespiratory coupling in health and disease. Finally, it can enable the online analysis of the respiratory pattern changes in the monitored patients, which might be vital in patients with COVID or other life-threatening conditions.

Unfortunately, studies have not considered such a complex analysis of breathing patterns and their relationship with anthropomorphic health indicators. Although reports concerning lung/asthmatic breath classification exist [56, 57], they identify respiratory patterns employing recorded signals and machine learning following conventional chest auscultation with a stethoscope rather than taking a plethora of health indicators in the analysis. In their study, Göğüş et al. classified inhalation and exhalation sound signals of 11 persons based on features derived from DWT and wavelet packet transform (WPT) signals along with an artificial neural network (ANN). ANN was used to classify respiratory sounds into four classes: normal, mild asthma, moderate asthma, and severe asthma [56]. The obtained classification accuracies are high; however, the number of signals used is too small to provide meaningful observation. Kandaswamy et al. [57] classified 126 signal samples. Still, their focus was on several lung sound categories: normal, wheeze, crackle, squawk, stridor, or rhonchus, so it is difficult to estimate whether these results would be held when applied to a larger dataset. Consequently, a direct comparison of the results obtained is not possible.

Future studies will be directed towards a twofold aim. First, we would like to improve the process of detecting pauses and apneas. This approach will help the expert assess the breathing pattern. Furthermore, it might be treated as a pre-processing stage supporting further analyses. One of the planned approaches will comprise modified VAD (voice activity detection) algorithms. VAD algorithms are a critical part of speech processing, recognition, and coding systems. Their operation principle is to detect and separate fragments of silence (pauses) and regions containing speech. Attempts at respiratory pattern assessment using such algorithms can be found in the literature; however, the experiments were limited to signals recorded using a microphone [28, 58, 59]. We believe that modifying the VAD algorithms will allow their application to analyze the signals coming from the respiratory belt. Moreover, in future studies, we would like to follow the second aim by employing other techniques, such as the rough-fuzzy approach, to identify the best way to analyze the gathered data, as some of the parameters acquired need creating membership functions and fuzzification.
